# Older Adults’ Receptivity to Spousal Health Support: A Qualitative Analysis

**DOI:** 10.1093/geroni/igaa057.2237

**Published:** 2020-12-16

**Authors:** Amy Rauer, Ruth Palan Lopez, Ashley Pigg

**Affiliations:** 1 The University of Tennessee, Knoxville, Knoxville, Tennessee, United States; 2 University of Tennessee, Knoxville, Knoxville, Tennessee, United States

## Abstract

Romantic partners play a powerful role in promoting each other’s well-being, particularly later in life. Although there is a robust literature documenting how spouses tend to respond to each other’s health issues (e.g., support, control), there has been a surprising dearth of studies examining how receptive spouses are to these overtures. To reveal how older couples react to each other’s health support attempts, we used a qualitative approach to analyze transcripts of 64 older, married, heterosexual couples observed discussing each other’s health issues. Thematic analysis revealed older couples responded to spouses’ health support strategies in four different ways: 1) listening (individuals actively listened); 2) accepting (individuals welcomed spousal efforts); 3) deflecting (individuals redirected conversation away from health); and 4) rejecting (individuals dismissed and refused spousal intervention). Given the rise in late-life caregiving, identifying the very different responses that are evoked when partners intervene in each other’s health warrants further investigation. Part of a symposium sponsored by Dyadic Research on Health and Illness Across the Adult Lifespan Interest Group.

